# Identification of Anti-EGFR and Anti-ErbB3 Dual Variable Domains Immunoglobulin (DVD-Ig) Proteins with Unique Activities

**DOI:** 10.1371/journal.pone.0124135

**Published:** 2015-05-21

**Authors:** Jinming Gu, Jinsong Yang, Qing Chang, Zhihong Liu, Tariq Ghayur, Jijie Gu

**Affiliations:** 1 AbbVie Bioresearch Center, R&D, Worcester, Massachusetts, 01605, United States of America; 2 Cancer Research, R&D, AbbVie Inc., North Chicago, Illinois, 60064, United States of America; National Health Research Institutes, TAIWAN

## Abstract

Epidermal growth factor receptor (EGFR) and receptor tyrosine-protein kinase 3 (ErbB3) are two well-established targets in cancer therapy. There is significant crosstalk among these two receptors and others. To block signaling from both EGFR and ErbB3, we generated anti-EGFR and anti-ErbB3 DVD-Ig proteins. Two DVD-Ig proteins maintained the functions of the combination of the two parental antibodies. The DVD-Ig proteins inhibit cell signaling and proliferation in A431 and FaDu cells while half DVD-Ig proteins lost proliferation inhibition function. Interestingly, in the presence of β-Heregulin (HRG), the DVD-Ig proteins show synergies with respect to inhibiting cell proliferation. The DVD-Ig proteins downregulate EGFR protein expression in the presence of HRG, which may be due to receptor internalization. Furthermore, the DVD-Ig proteins remarkably disrupt β-Heregulin binding to FaDu cells.

## Introduction

Receptor tyrosine kinase (ErbB) family sigaling plays key roles in development and disease [1]. In particular, disregulation of ErbB signaling is one of the most frequent events in solid tumor progression [2]. Among ErbB family members, EGFR, ErbB2, and ErbB3 have been extensively studied. Targeted therapies against EGFR, ErbB2, or ErbB3 are under clinical development or have been approved by the FDA. Cetuximab is a chimeric anti-EGFR antibody that was approved by the FDA in 2004 and has been used to treat a wide variety of human tumors [3–5]. MM121 is an extensively studied fully human anti-ErbB3 antibody that has been developed by Merrimack Pharmaceuticals [6–8]. MM121 was shown to inhibit cancer cell signaling and proliferation in vitro and tumor growth in vivo and is currently in Phase II human clinical trials [6–8].

The major limitations of current anti-EGFR therapies are toxicity and drug resistance. There is some evidence that anti-EGFR therapy drug resistance is due partially to amplification of ErbB3 signaling [9]. This observation has led to the hypothesis that concurrently blocking EGFR and ErbB3 pathways may have superior activities compared to blocking with single antibodies. Preclinical xenograft tumor models were used to demonstrate a “two-in-one” antibody against EGFR and ErbB3 called MEHD7945A has better activities than the parent antibodies alone and has similar activity to the combination of the two parent antibodies alone, in addition to with lower cyno-toxicity [10]. MEHD7945A has inhibitory activities against EGFR- and ErbB3- mediated signaling *in vitro* and *in vivo* [10]. This bispecific antibody is currently undergoing phase II clinical evaluation in patients with kRAS wild-type metastatic colorectal cancer.

While certain two-in-one antibodies have shown some success in preclinical development, this platform may have certain limitations. First, it is time consuming to generate certain two-in-one antibodies. One has to develop an antibody against one target and then design a library to screen against the second target. Second, two-in-one antibodies may function as the combination of the two single arm antibodies with restricted avidity as a consequence of its structure.

We have developed a bispecific platform, dual variable domain immunoglobulin (DVD-Ig) molecules [11]. Certain DVD-Ig proteins maintain drug-like properties similar to mAbs and can be designed to target two different targets or two different epitopes on the same target. DVD-Ig technology allows for the combination of immunoglobulin variable domain sequences into the DVD-Ig framework in different configurations. We hypothesized that we could use two immunoglobulin variable domain sequences specific for EGFR and ErbB3, respectively, to create DVD-Ig molecules to explore whether we can capture the combination effect of the two single antibodies or may go beyond the mechanisms of two combined antibodies.

Here we described the generation and characterization of anti-EGFR/ErbB3 DVD-Ig proteins. We found that the anti-EGFR/ErbB3 DVD-Ig proteins retain the activities of both parental antibodies in binding assays. Interestingly, the anti-EGFR/ErbB3 DVD-Ig proteins inhibit A431 and FaDu cell proliferation and cell signaling with some synergistic activities. We further studied the mechanism of action of these DVD-Ig proteins.

## Results

### Generation of anti-EGFR and anti-ErbB3 DVD-Ig proteins

**Fig 1:**
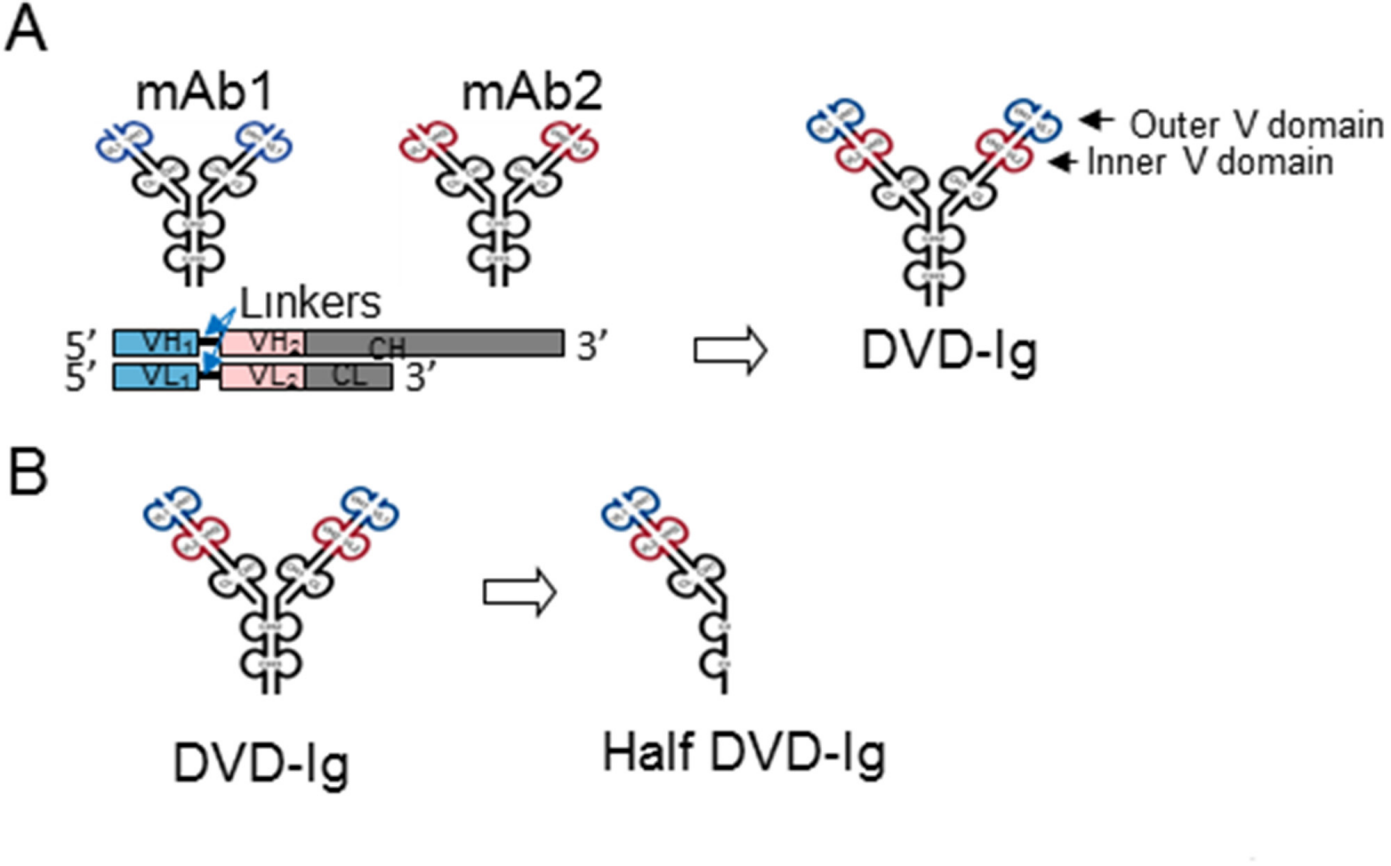
Generation of anti-EGFR and anti-ErbB3 DVD-Ig and half DVD-Ig proteins. (A) DVD-Ig molecules were generated by linking the variable domains mAb1 and mAb2, using various orientations of the two variable domains and linkers. (B) Half DVD-Ig molecules were generated with two mutations (C220S and C226S) in the hinge region and four mutations in the C_H_3 region (P395A, F405R, Y407R, and K409D).

To test whether we could capture the combination effects of an anti-EGFR mAb and an anti-ErbB3 mAb via the DVD-Ig platform, we utilized their variable domains with human IgG1/κ constant domains. DVD-Ig molecules were generated using various orientations of the two variable domains and linkers (see Materials and Methods for details) (Table 1, Fig 1, and data not shown). We then transiently expressed the DVD-Ig proteins in 293 cells and purified them to homogeneity with Protein A columns. We found that some DVD-Ig proteins showed relatively high expression levels (>5mg/L) and low levels of aggregation (Table 1 and data not shown), which indicated that they were suitable for further characterization.

**Table 1:** Characterization of EGFR and ErbB3 binding of anti-EGFR-ErbB3 DVD-Igs.

Antibodies	Outer domain	Inner domain	Linker	Aggregates	EC50 (EGFR) nM	EC50 (ErbB3) nM
anti-EGFR	N/A	N/A	N/A	<10%	1.2	N/A
anti-ErbB3	N/A	N/A	N/A	<10%	N/A	0.8
anti-EGFR/ErbB3 bsAb	N/A	N/A	N/A	<10%	1.3	5.7
DVD1	anti-EGFR	Anti-ErbB3	L-S	<10%	1.7	3.2
DVD2	anti-EGFR	Anti-ErbB3	S-L	<10%	1.4	2.1

### EGFR and ErbB3 binding characterization of anti-EGFR/ErbB3 DVD-Ig proteins

We then tested the binding of these DVD-Ig proteins to EGFR or to ErbB3 via ELISA. The results showed that DVD1 and DVD2 proteins maintain the binding affinity of the parental mAbs (Table 1).

### Anti-EGFR and anti-ErbB3 DVD-Ig proteins inhibit cancer cell proliferation

**Fig 2:**
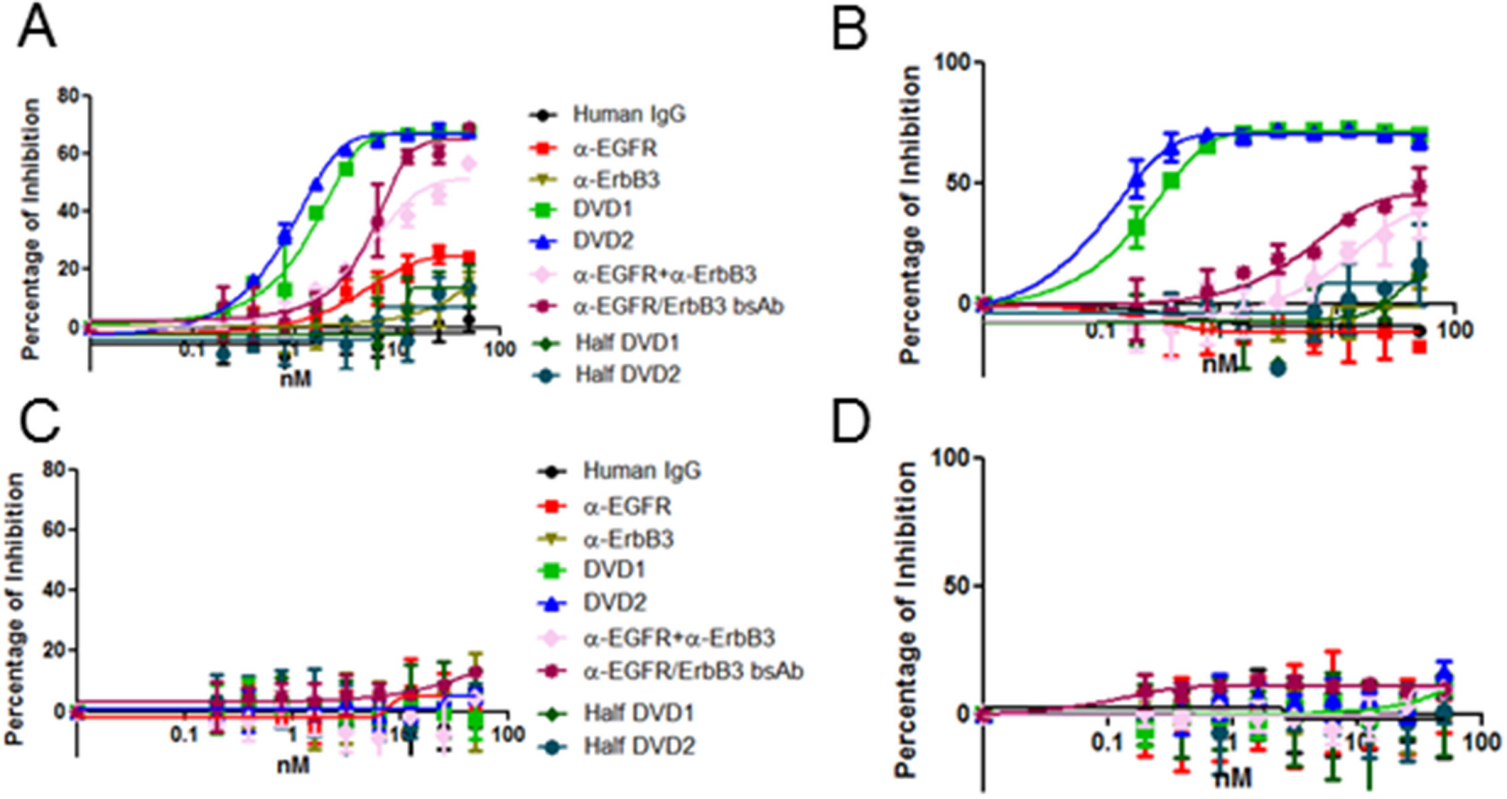
Anti-EGFR/ErbB3 DVD-Ig proteins inhibit cell proliferation. (A) A431, (B) FaDu, (C) LNCaP, and (D) PC3 cells were treated with different dosages of anti-EGFR/ErbB3 DVD-Ig proteins, anti-EGFR/ErbB3 half DVD-Ig proteins anti-EGFR and anti-ErbB3 mAbs alone, anti-EGFR and anti-ErbB3 mAbs in combination, or the bsAb for 10 days. After 10 days, cells were fixed and stained with with Crystal Violet. Cell proliferation was measured with Crystal Violet staining. The error bars indicate standard deviation from the mean.

To test whether the anti-EGFR/ErbB3 DVD-Ig proteins could inhibit cancer cell proliferation, we screened the DVD-Ig proteins, in the presence or absence of HRG, using a panel of human cancer cell lines via cell proliferation assay (Fig 2 and data not shown). We found that the anti-EGFR/ErbB3 DVD-Ig proteins showed the best proliferation inhibition in A431 and FaDu cells, both of which express relatively high levels of EGFR and ErbB3 (S1 Fig). In contrast, the anti-EGFR/ErbB3 DVD-Ig proteins showed no proliferation inhibition in LNCaP and PC3 cells, both of which express relatively low levels of EGFR and ErbB3 (S1 Fig). In both A431 and FaDu cells, both DVD1 and DVD2 proteins significantly inhibited cell proliferation to a level similar to that for the combination of the two single antibodies at maximum dosage (Fig 2). Interestingly, DVD1 and DVD2 proteins have lower IC50 and better maximum inhibition compared to parental mAbs alone, the anti-EGFR/ErbB3 bispecific Ab (bsAb), or in combination in the presence of HRG (Fig 2). However, in the absence of HRG, DVD1 and DVD2 proteins only have lower IC50 compared to parental mAbs alone or in combination (data not shown).

We then asked whether the avidity effect of the DVD-Ig molecules is critical for the inhibition of cell proliferation. To answer this, we generated and tested half DVD1 and half DVD2 proteins, which only retain the single arm of a DVD-Ig protein to eliminate avidity effects. Interestingly, we found that half DVD1 and half DVD2 molecules have lost their ability to inhibit cell proliferation (Fig 2), suggesting that the avidity effect of the DVD-Ig molecules maybe critical for that function.

### Anti-EGFR and anti-ErbB3 DVD-Ig proteins inhibit cell signaling

**Fig 3:**
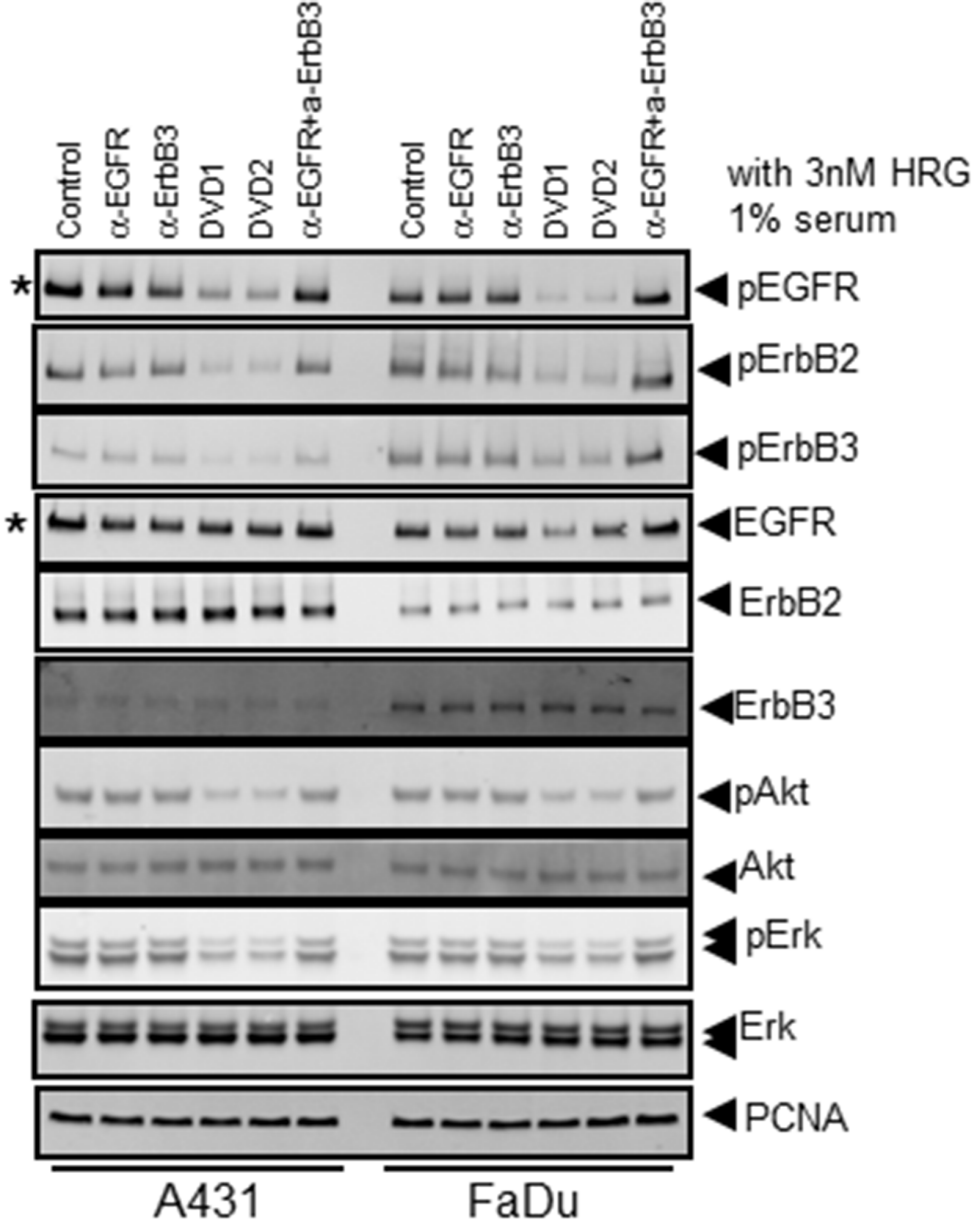
Anti- EGFR/ErbB3 DVD-Ig proteins in cell signaling assay. A431 and FaDu cells were cultured in medium containing 3nM HRG and 1% serum were treated with 100nM anti-EGFR and anti-ErbB3 mAbs alone, anti-EGFR and anti-ErbB3 mAbs in combination, the bsAb or anti-EGFR/ErbB3 DVD-Ig proteins for 24 hours. Cells were then harvested and lysed for Western blot. * indicates that 10% of sample lysates were used for A431 cells.

We then tested whether the anti-EGFR/ErbB3 DVD-Ig proteins could inhibit cell signaling. EGFR and ErbB3 form heterodimers on the cell surface upon ligand binding to induce downstream signaling of multiple pathways including PI3K/AKT and Ras/MAPK. A431 or FaDu cells were treated with the anti-EGFR and anti-ErbB3 single antibodies or anti-EGFR/ErbB3 DVD-Ig proteins in the presence of HRG for 24 hours. The results showed that DVD1 and DVD2 proteins significantly inhibit cell signaling, especially pEGFR, pErbB2, pErbB3, and pAkt, in both A431 and FaDu cells (Fig 3). In another assay, we serum starved A431 or FaDu cells for 24 hours and then pretreated with the anti-EGFR and anti-ErbB3 single antibodies or anti-EGFR/ErbB3 DVD-Ig proteins. After HRG stimulation for 10 minutes, cells were harvested. Under this transient stimulation condition, DVD1 and DVD2 proteins inhibit cell signaling at the similar level as to anti-EGFR and anti-ErbB3 single antibodies, combination, and the bsAb (S2 Fig). Taken together, these results suggest that DVD1 and DVD2 proteins have longer cell signaling inhibition effects compared to the anti-EGFR and anti-ErbB3 single antibodies, combination, and the bsAb.

### Anti-EGFR/ErbB3 DVD-Ig proteins mechanism of actions (MOAs)

**Fig 4:**
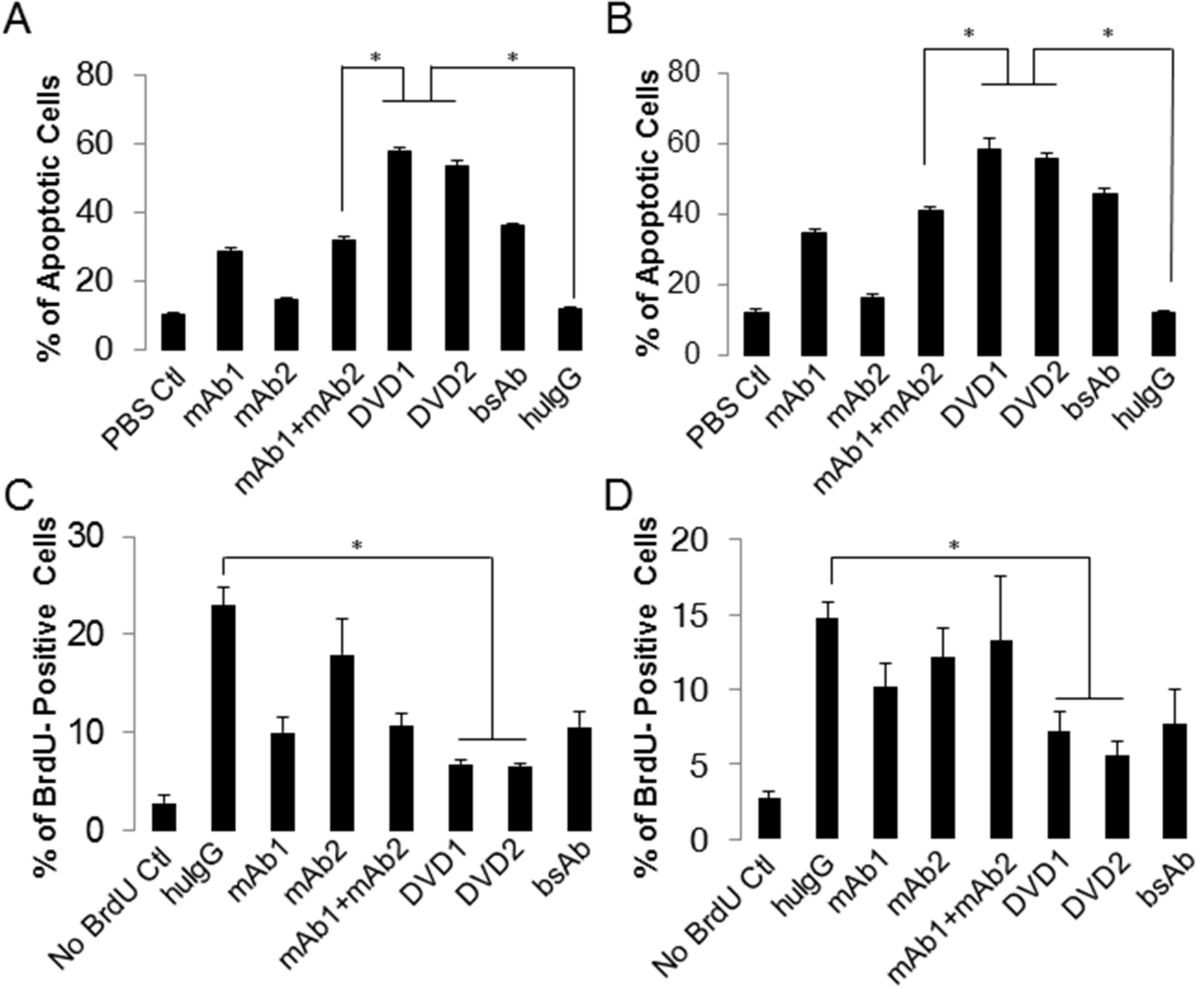
Anti- EGFR/ErbB3 DVD-Ig proteins induce apoptosis and arrest cell cycle. An apoptosis assay was used to analyze (A) A431 or (B) FaDu cells after DVD-Ig proteins, mAbs, or combination treatment. Apoptotic (Annexin V-positive) cells were quantitated via FACS. A BrdU-incorporation assay was used to analyze (C) A431 or (D) FaDu cells after DVD-Ig proteins, mAbs, or combination treatment. BrdU-positive cells were quantitated via FACS. Three independent experiments with triplicates were performed. The error bars indicate standard deviation from the mean. p value was calculated via student T-test. *p<0.05.

To determine whether anti-EGFR/ErbB3 DVD-Ig proteins exhibit better inhibition of A431 and FaDu cell proliferation, we performed apoptosis and cell cycle analysis. Interestingly, the results show that a 72-hour anti-EGFR/ErbB3 DVD-Ig protein (DVD1 or DVD2) treatment significantly induced apoptosis (both early and late apoptotic cells; S3 Fig) in both A431 and FaDu cells (Fig 4A and 4B) more than the combination of mAb1 and mAb2. In the cell cycle assay, anti-EGFR/ErbB3 DVD-Ig proteins DVD1 and DVD2 showed a significant decrease in BrdU-positive cells in both A431 and FaDu cells (Fig 4C and 4D). In contrast, we did not observe the same phenomenon in LNCaP or PC3 cells (S4 Fig). Together, these data are consistent with what observed in the cell proliferation assay: anti-EGFR/ErbB3 DVD-Ig proteins DVD1 and DVD2 induce more apoptosis and arrest more cells in the cell cycle in both A431 and FaDu cells, which resulted in better proliferation inhibition; while anti-EGFR/ErbB3 DVD-Ig proteins DVD1 and DVD2 have no such effects in LNCaP or PC3 cells.

### Anti-EGFR/ErbB3 DVD-Ig protein shows enhanced internalization

When we performed the cell signaling assay, we noticed that DVD1 and DVD2 proteins could down-regulate EGFR protein levels (Fig 3 and data not shown). We hypothesized that DVD1 and DVD2 proteins induce cell surface EGFR internalization, and then are targeted for degradation.

**Fig 5:**
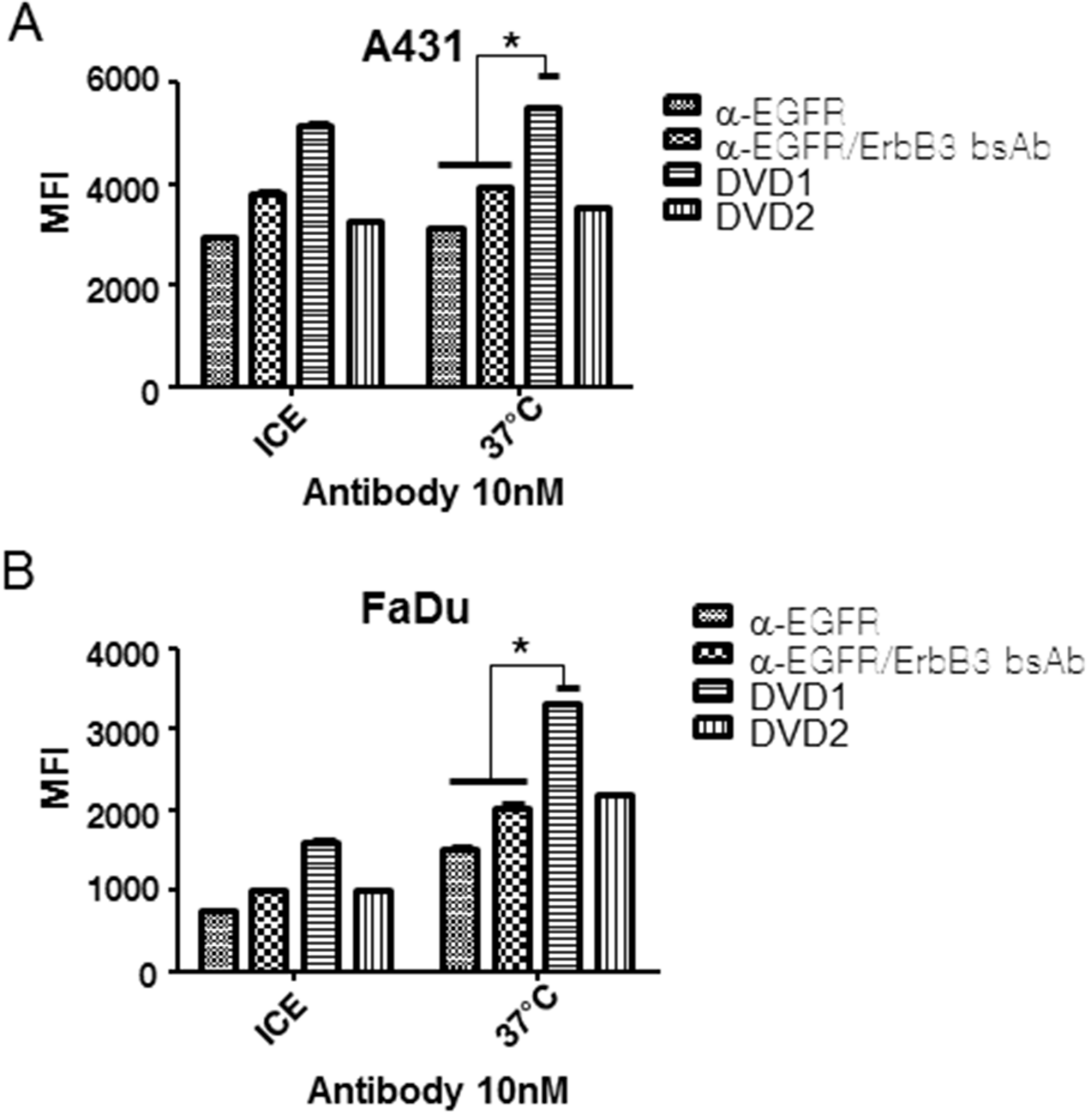
Anti- EGFR/ErbB3 DVD-Ig protein internalization. (A) A431 and (B) FaDu cells were treated with 10nM pHRodo-labeled anti-EGFR mAb alone, the bsAb, or anti-EGFR/ErbB3 DVD-Ig proteins on ice or at 37°C for 2 hours in PBS. Fluorescence intensity was measured via FACS. The error bars indicate standard deviation from the mean. p value was calculated via student T-test. *p<0.05.

To test this hypothesis, we labeled mAb and DVD-Ig proteins with pHRodo, which is a fluorescence dye that only signals at low pH (intracellular). When cells were treated with the pHRodo-labeled mAbs or DVD-Ig proteins, we found that DVD1 showed significantly enhanced internalization compared to the mAbs or the bsAb at 37°C (Fig 5A and 5B).

We observed strong internalization of anti-EGFR mAb1, bsAb, DVD1, and DVD2 proteins in A431 cells after 2-hour incubation on ice. We further tested the internalization after 10-minute incubation on ice. The results show anti-EGFR mAb1, bsAb, DVD1, and DVD2 proteins could be internalized even after 10-minute incubation on ice (S5A Fig). We next confirmed the internalization via confocal microscopy, which showed that anti-EGFR mAb1, bsAb, DVD1, and DVD2 proteins could be internalized into A431 cells even after 10-minute incubation on ice (S6A and S6C Fig and data not shown).

In contrast, DVD1 does not enhance internalization in PC3 cells after 10-minute or 2-hour incubation at 37°C (S5B and S5C Fig). Confocal microscopy results showed that in PC3 cells anti-EGFR mAb1, bsAb, DVD1, and DVD2 proteins have limited internalization after 4-hour incubation 37°C (S6F Fig and data not shown).

### Anti-EGFR/ErbB3 DVD-Ig protein shows enhanced HRG ligand blocking

It is known that HRG binds to ErbB3 with low affinity. However, upon HRG binding ErbB3 can form heterodimers with EGFR or ErbB2 to form a high affinity complex [12]. Like mAbs, DVD-Ig molecules have avidity effects. We reasoned that anti-EGFR/ErbB3 DVD-Ig proteins may bind to cell surface EGFR and ErbB3 and exert an avidity effect so that a minimal amount of HRG could bind to ErbB3, thereby blocking HRG-induced signaling more efficiently than mAbs specific to ErbB3.

**Fig 6:**
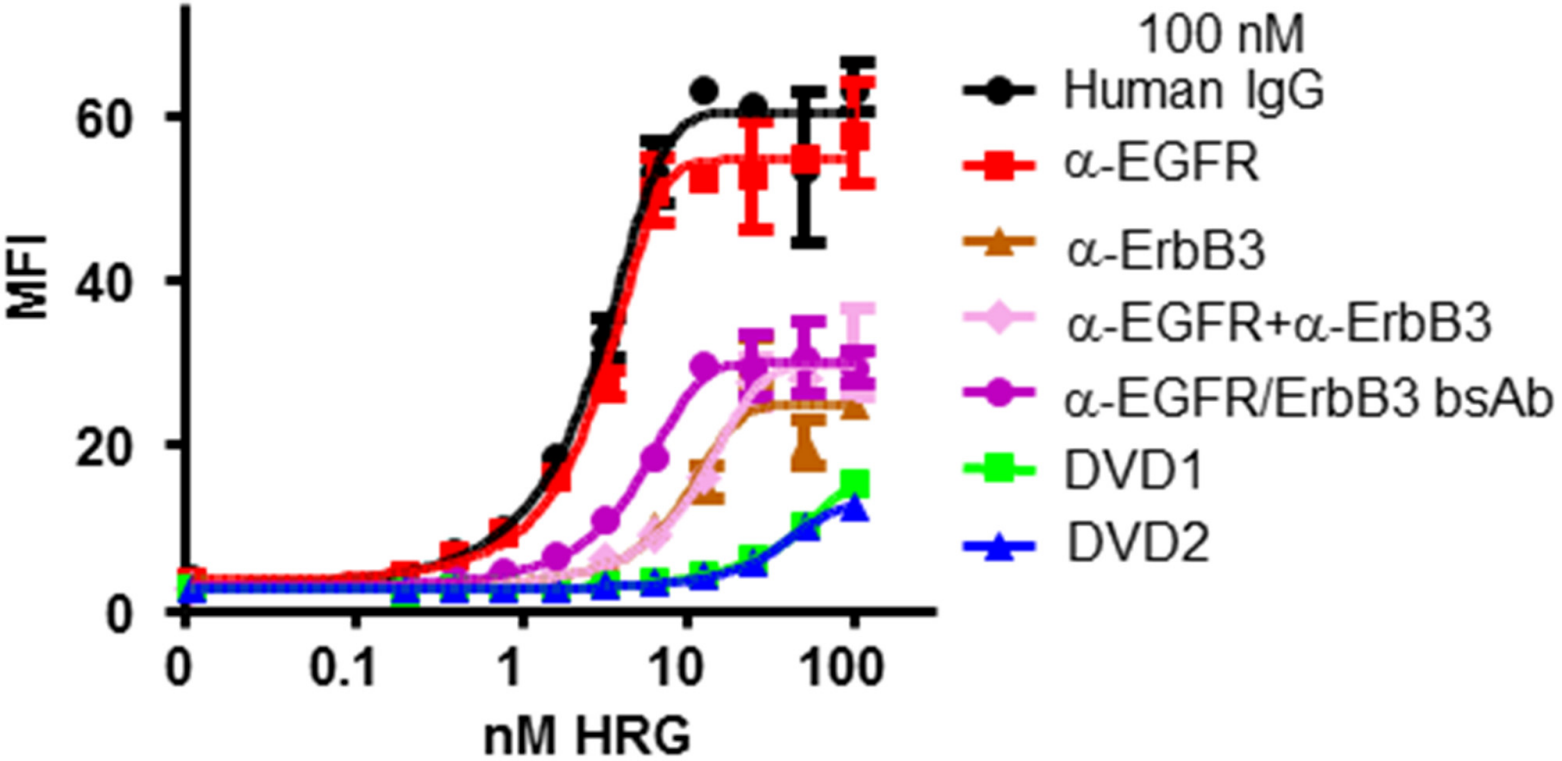
DVD-Ig proteins inhibit HRG binding to FaDu cells. Serial dilutions of HRG were incubated with 100 nM anti-EGFR and anti-ErbB3 mAbs alone, anti-EGFR and anti-ErbB3 mAbs in combination, the bsAb, or anti-EGFR/ErbB3 DVD-Ig proteins. HRG binding affinity to FaDu cells was measured via FACS. MFI: median fluorescence intensity. The error bars indicate standard deviation from the mean.

To test this hypothesis, we analyzed the binding affinity of Alexa488-labeled HRG to FaDu cells in the presence of the anti-EGFR and anti-ErbB3 mAbs alone, the anti-EGFR and anti-ErbB3 mAbs in combination, and the anti-EGFR/ErbB3 DVD-Ig proteins. The results showed that both DVD1 and DVD2 proteins could block HRG binding to FaDu cells more efficiently (Fig 6). For example, when incubated with 10nM HRG, 100nM DVD1 and DVD2 could block >90% of HRG binding to the cells, while 100nM anti-ErbB3 mAb, the bsAb, or the combination could only block ~75% of HRG binding to the cells (Fig 6).

In contrast, neither DVD1 nor DVD2 proteins could block HRG binding to PC3 cells (S7 Fig).

## Discussion

The ErbB family members have been extensively studied and identified as potential cancer therapeutic targets [2]. Since they form heterodimers and induce signal crosstalk, the ErbB family members have become drug targets for bi-specific antibodies. For example, the anti-ErbB2/ErbB3 bsAb MM111 (Merrimack Pharmaceuticals) is in Phase II clinical trials and the Genentech two-in-one antibody is in Phase II clinical trials, and both have shown some positive clinical indications.

Here we present evidence that anti-EGFR/ErbB3 DVD-Ig proteins maintain the function of the combination of the two parental mAbs. In certain circumstances, the anti-EGFR/ErbB3 DVD-Ig proteins show synergies that are not captured by the combination of the two parental mAbs, which is likely due to an avidity effect. Taking into consideration that generating DVD-Ig molecules would take relatively shorter time if two known mAb sequences are used, our study proves that DVD-Ig technology is a powerful platform to generate bi-specific immunoglobulins as therapeutics.

One question still remains, why DVD1 protein but not DVD2 protein enhances internalization. The difference between DVD1 protein and DVD2 protein is that DVD1 has long linker in heavy chain and short linker in light chain while DVD2 protein has short linker in heavy chain and long linker in light chain. We hypothesize that this subtle difference may cause the different conformational change upon binding to the cell surface receptors, which may trigger the enhanced internalization. More DVD proteins with similar linker combination need to be generated to test this hypothesis.

Interestingly, the anti-EGFR/ErbB3 DVD-Ig proteins block HRG binding to the FaDu cells with much greater potency than the combination of two mAbs and the bsAb. One of the main functions of HRG is to induce EGFR/ErbB3 heterodimer formation to activate downstream signals. It is possible that due to the unique structure and rigidity of the anti-EGFR/ErbB3 DVD-Ig proteins, they could bind one heterodimer complex with one arm so that the conformation of the complex has been changed to one that is not able to efficiently bind HRG, which could not be achieved by the combination of two mAbs or the bsAb.

Our results indicate that cell lines with high levels of EGFR and ErbB3 respond well to the anti-EGFR/ErbB3 DVD-Ig proteins treatment compared to those cells lines that express low levels of EGFR and ErbB3. In addition, we only observed the synergistic effects of anti-EGFR/ErbB3 DVD-Ig proteins in the presence of HRG. Several studies have suggested that HRG is overexpressed in human breast, ovarian, and prostate cancers [13–15], while blocking HRG binding to the tumor cell surface may have therapeutic significance. Our results suggest that human cancer patients with relatively high levels of EGFR, ErbB3, and HRG are the potential patient population of the anti-EGFR/ErbB3 DVD-Ig protein treatment.

Recently, we presented data showing anti-ErbB2 DVD-Ig proteins have unique activities in N87 cells, wherein some DVD-Ig proteins have agonist activities while the others have antagonist activities (similar to the combination of the two parental mAbs) [16], suggesting that DVD-Ig technology can create agonist effects out of two antagonist parental mAbs. In comparison, here we presented data showing some anti-EGFR/ErbB3 DVD-Ig proteins obtain better activities than the combination of the two parental mAbs, suggesting that DVD-Ig technology can create some additive if not synergistic effects that cannot be acquired by simply combining the two single mAbs.

With bi-specific immunoglobulins becoming one of the most extensively investigated areas in protein-based therapeutics, DVD-Ig technology holds great promise in bringing novel drugs to clinic in a more time-efficient manner. Combined with high-throughput mAb combination screening, DVD-Ig molecules could be generated, screened, and scaled-up to meet pharmaceutical pipeline development needs.

## Materials and Methods

### Construction, expression and purification of anti-EGFR/ErbB3 DVD-Ig molecules

Anti-EGFR/ErbB3 DVD-Ig molecules were generated as described previously [11,17]. Briefly, the VH and VL sequences (GenBank: GM685462.1) of an anti-EGFR mAb and the VH and VL sequences of an anti-ErbB3 mAb MM121 were linked with short (ASTKGP) or long (TVAAPSVFIFPP) linkers and expressed with human IgG1 heavy chain and Iκ light chain constant domains. The anti-EGFR/ErbB3 bsAb were generated from published MEHD7945A sequences (GenBank: GI:354459522 and GI:354459521). For half-DVD1 and half-DVD2, the VH and VL sequences of DVD1 or DVD2, respectively, were PCR cloned into a half DVD-Ig molecule expression construct, which was built with two mutations (C220S and C226S) in the hinge region and four mutations in the C_H_3 region (P395A, F405R, Y407R, and K409D) [18]. The plasmids encoding the HC and LC of each construct were transiently expressed in human embryonic kidney 293 cells (American Type Culture Collection (ATCC), VA) and purified using protein A chromatography (Pierce, IL) according to the manufacturer's instructions with yield >5mg/L. The resulting half-DVD-Ig proteins contain only a single arm of a full-length DVD-Ig protein as confirmed by size exclusion chromatography (SEC). All DVD-Ig proteins and mAbs were confirmed to be <10% aggregates by size exclusion chromatography (SEC). All DVD-Ig proteins and mAbs are stable at 4°C for at least 1 month. In all the assays, monoclonal antibodies or DVD-Ig proteins were compared at the same molarity as in combination, e.g., 10nM anti-EGFR mAb was compared to 10nM anti-ErbB3 mAb, 10nM anti-EGFR mAb +10nM anti-ErbB3 mAb, or 10nM DVD-Ig proteins.

### Cell lines and cell culture conditions

A431, FaDu, LNCaP, and PC3 cells were obtained from the ATCC (VA). All cells were maintained sub-confluent in DMEM medium supplemented with 10% fetal bovine serum (FBS), 50 units/mL penicillin, and 50 μg/mL streptomycin at 37°C and 5% CO_2_.

### Binding affinity measurements

Antibodies or DVD-Ig proteins binding affinity were measured with enzyme linked immunosorbent assays (ELISAs). Briefly, plates were coated with 0.5μg/ml of anti-his antibody (Invitrogen, CA) in carbonate buffer at 4°C overnight. After washing with PBS and blocking with Superblock (Pierce, CA) at room temperature (RT) for one hour, 100 μl of 0.1 mg/ml His-tagged EGFR or ErbB3 proteins (Invitrogen, CA) were added in PBS with1% BSA for one hour at RT. After washing with PBS, gradient-diluted antibodies or DVD-Ig proteins at the indicated concentrations were added for one hour at RT and captured antibodies or DVD-Ig proteins were detected by HRP-conjugated goat-anti-human antibodies (Jackson Immunoresearch, PA)

### Immunoblot analysis

Low passage log-phase cells were plated into 6-well plates and incubated overnight. Cells were incubated with 1ml 100nM antibodies or DVD-Ig proteins for 24 hours in the presence of 3nM HRG. Cells were then harvested and lysed with RIPA buffer (Sigma-Aldrich, MO) supplemented with protease and phosphatase inhibitor cocktail tablet according to the manufacturer's instructions (Roche Diagnostics, IN). Cell lysate proteins were resolved by SDS-PAGE and immunoblots were probed with antibodies against phosphorylated EGFR(Tyr^1068^; Cat#3777), ErbB2(Tyr^1221/1222^; Cat#2243), and ErbB3(Tyr^1197^; Cat#4561) (Cell Signaling, MA), EGFR (NeoMarker,Thermo Fisher, CA), ErbB2 and ErbB3 (Cell Signaling, MA), phosphorylated extracellular signal-regulated kinase (pERK) 1/2 (Thr^202^/Tyr^204^; Cat#4370) (Cell Signaling, MA), ERK1/2, AKT (Santa Cruz, CA), ErbB2 (Cell Signaling, CA), phosphorylated AKT (pAKT; Ser^473^; Cat#4060) (Cell Signaling, MA), followed by incubation with IRDye 700 conjugated goat anti-mouse and IRDye 800 conjugated goat anti-rabbit (LI-COR, NE). Total protein was normalized with anti-PCNA (Santa Cruz, CA) followed by IRDye680 CW conjugated goat anti-mouse (LI-COR, NE). Blots were visualized using an Odyssey Imaging system (LI-COR, NE).

### Cell proliferation assays

Low passage log-phase cells were seeded into 96-well plates at 1x10^3^ cells/well and incubated overnight in 1% serum. Antibodies or DVD-Ig proteins were added together with 3nM HRG as indicated above and cells were incubated for ten days. Following washing with PBS, cells were fixed with 4% formaldehyde (Sigma, MO), and stained with 0.1% crystal violet (Sigma, MO). Stained crystal violet was extracted with 10% acetic acid and quantitated at OD540.

### Apoptosis assay

Low passage log-phase A431 or FaDu cells (10,000/well) were seeded into 6-well plates and incubated overnight. Antibodies or DVD-Ig proteins were added at 100nM and apoptotic cells were counted for three days later by FITC-Annexin V (BD Biosciences, CA) and propidium iodide (Sigma-Aldrich, MO) labeling according to the manufacturer’s protocol. Annexin V-positive and propidium iodide-negative cells were counted as early apoptotic cells (Q3); Annexin V and propidium iodide double positive cells were counted as late apoptotic and necrotic cells (Q2). Together, Annexin V-positive cells were counted as apoptotic cells (Q2+Q3) (S3 Fig).

### BrdU-incorporation assay

Low passage log-phase A431 or FaDu cells (100,000/well) were seeded into 6-well plates and incubated overnight. Antibodies or DVD-Ig proteins were added at 100nM and cells were treated for two days. Cells were pulse-labeled with 10mM BrdU (BD Biosciences, CA) for 30 minutes at 37°C and then harvested and stained with BrdU-FITC (BD Biosciences, CA) according to the manufacturer’s protocol.

### Internalization assay

mAbs and DVD-Ig proteins were labeled with pHrodo red using the manufacturer’s protocol (Invitrogen, CA). Labeled mAbs and DVD-Ig proteins were quantitated with Nanodrop (Fisher, CA). Low passage log-phase A431 or FaDu cells were harvested with Accutase (Invitrogen, CA) and incubated with 10 nM labeled mAbs or DVD-Ig proteins on ice or at 37°C for 2 hours in PBS. Cells were washed and analyzed on a BD Fortessa (BD Biosciences, CA) with FlowJo (Tree Star, OR).

### HRG binding assay

HRG-ECD protein (R&D Systems, MN) was labeled with Alexa 488 using the manufacturer’s protocol (Invitrogen, CA). Low passage log-phase FaDu cells were harvested with cell dissociation buffer (Invitrogen, CA) and incubated with different concentrations of labeled HRG in the presence of 100nM antibodies or DVD-Ig proteins on ice for 45 minutes in PBS. Cells were washed with PBS and analyzed on a FACS Calibur (BD Biosciences, CA) with FlowJo (Tree Star, OR).

## Supporting Information

S1 Fig ErbB receptor expression. (TIF)

S2 Fig Anti- EGFR/ErbB3 DVD-Ig proteins in cell signaling assay. (TIF)

S3 Fig Apoptosis assay FACS gating. (TIF)

S4 Fig Anti- EGFR/ErbB3 DVD-Ig proteins do not induce apoptosis or arrest cell cycle in LNCaP or PC3 cells. (TIF)

S5 Fig Anti- EGFR/ErbB3 DVD-Ig protein internalization via FACS. (TIF)

S6 Fig Anti- EGFR/ErbB3 DVD-Ig protein internalization via confocal microscopy. (TIF)

S7 Fig DVD-Ig proteins inhibit HRG binding to FaDu cells. (TIF)
