# Supplementary figures and images for: Identification of Anti-EGFR and Anti-ErbB3 Dual Variable Domains Immunoglobulin (DVD-Ig) Proteins with Unique Activities
Source: PLoS One. 2015 May 21;10(5):e0124135. doi: 10.1371/journal.pone.0124135 (PMC4440733; doi:10.1371/journal.pone.0124135)

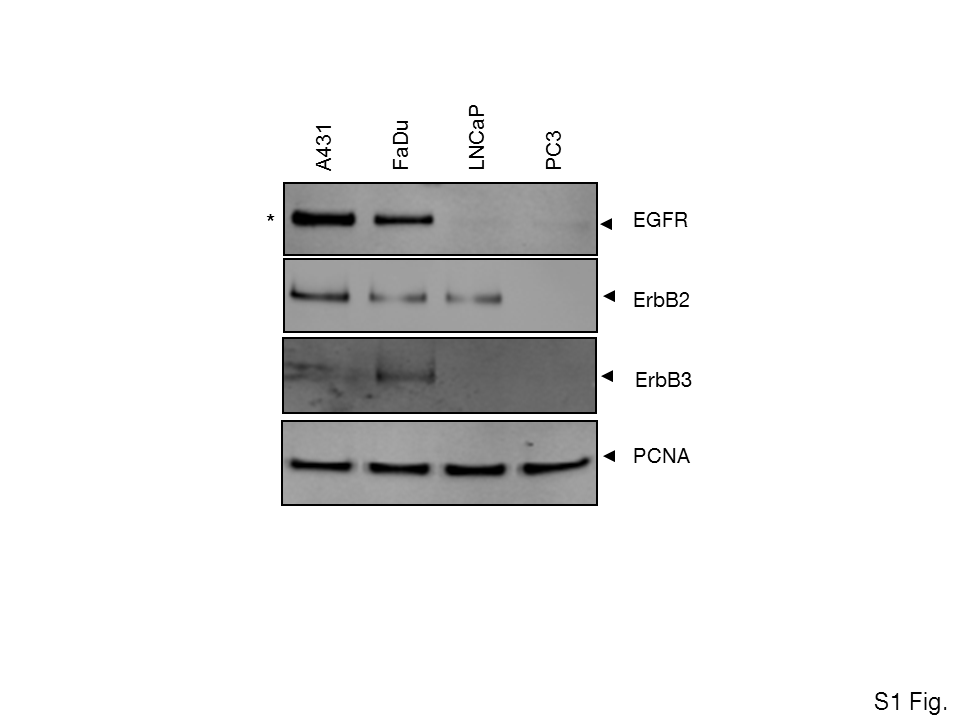

Supplement: S1 Fig — (TIF) [file pone.0124135.s001.TIF]

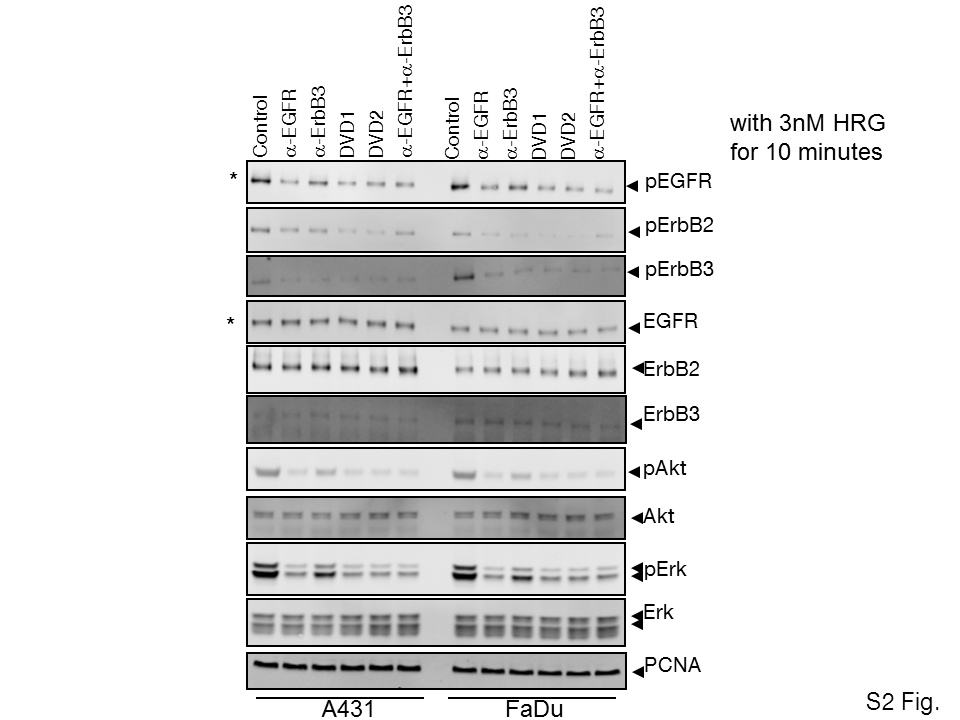

Supplement: S2 Fig — (TIF) [file pone.0124135.s002.TIF]

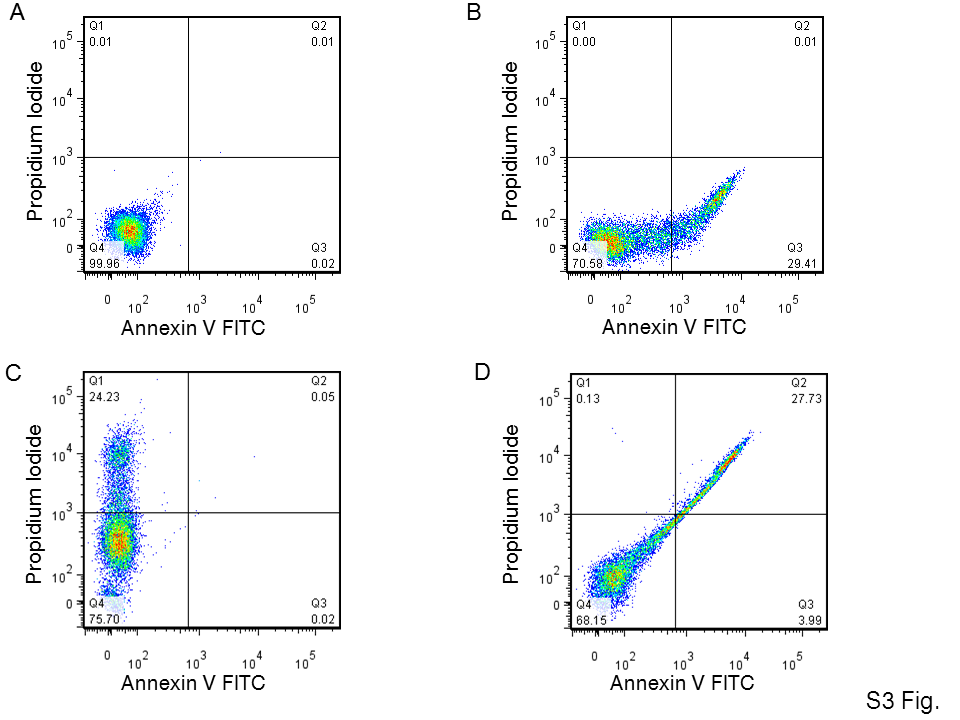

Supplement: S3 Fig — (TIF) [file pone.0124135.s003.TIF]

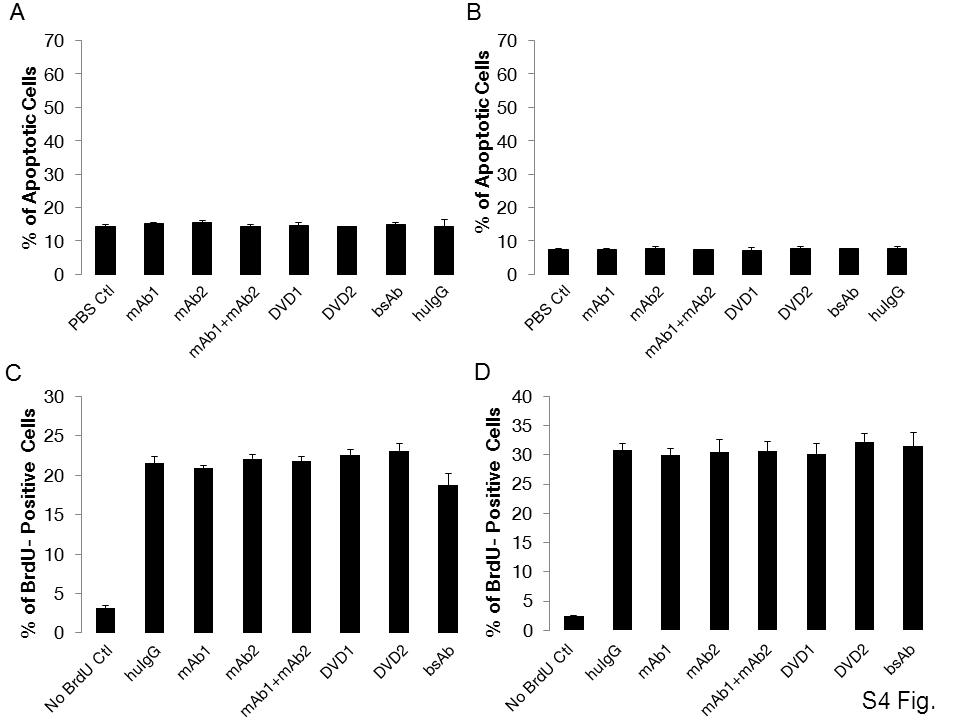

Supplement: S4 Fig — (TIF) [file pone.0124135.s004.TIF]

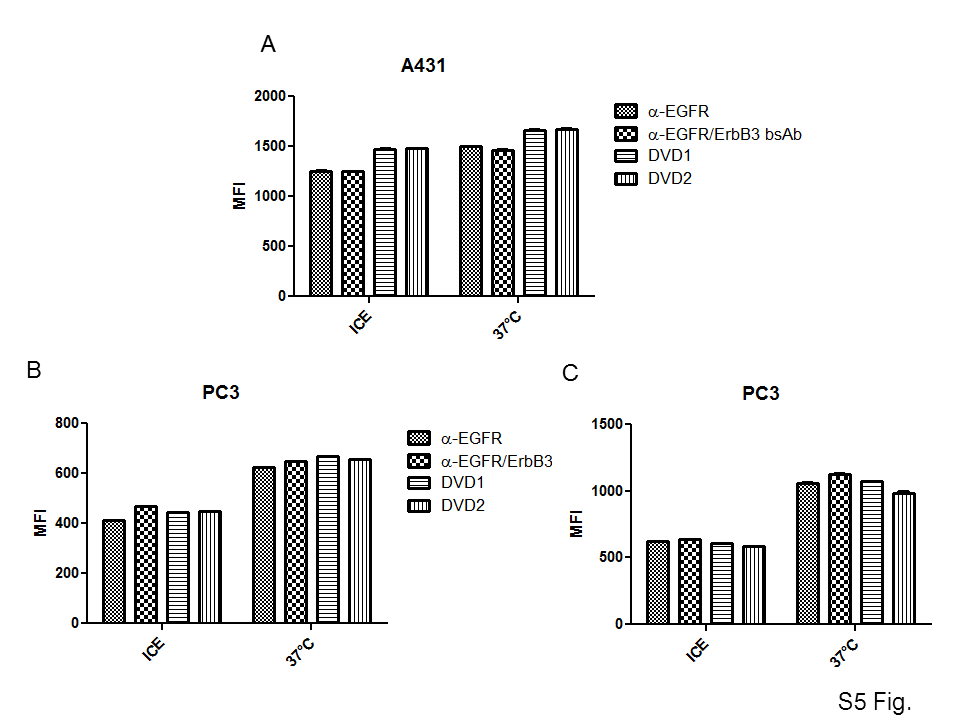

Supplement: S5 Fig — (TIF) [file pone.0124135.s005.TIF]

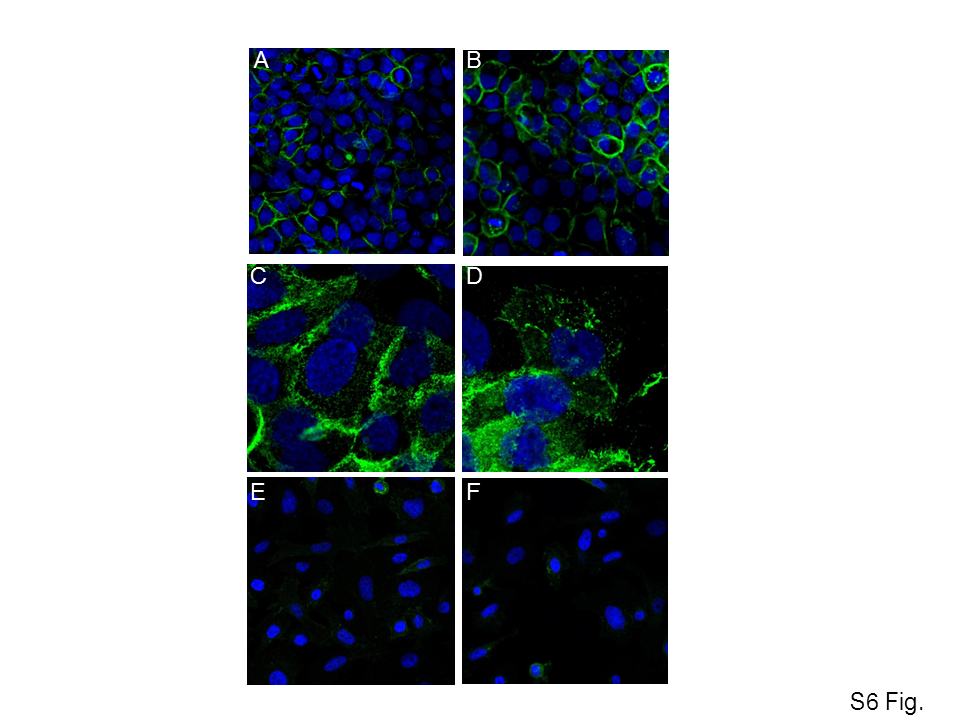

Supplement: S6 Fig — (TIF) [file pone.0124135.s006.TIF]

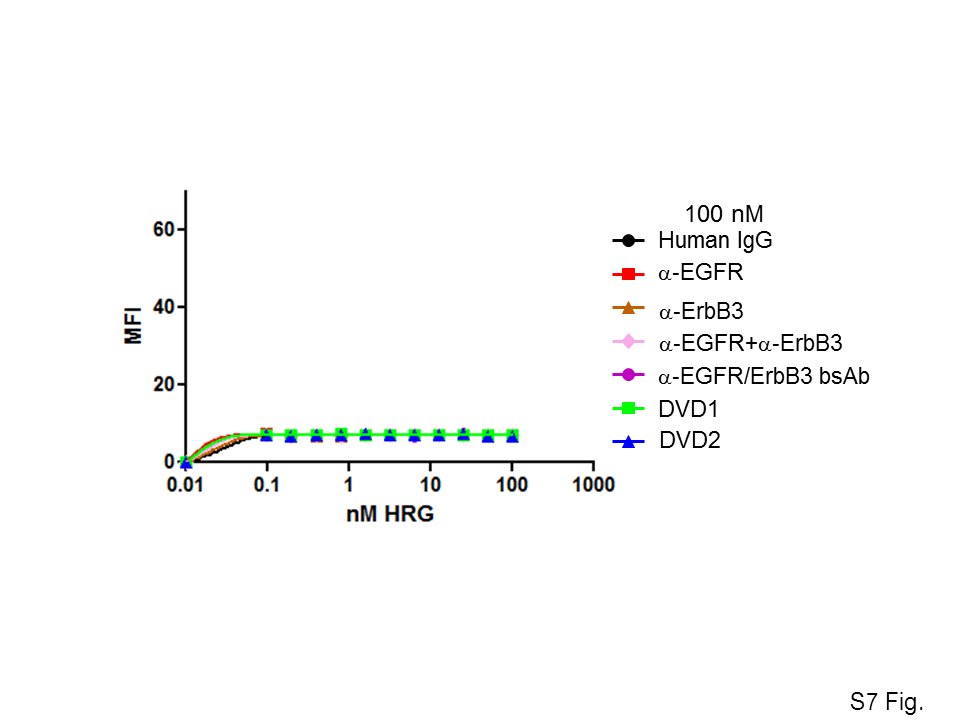

Supplement: S7 Fig — (TIF) [file pone.0124135.s007.TIF]
